# Novel design and development of *Centella Asiatica* extract - loaded poloxamer/ZnO nanocomposite wound closure material to improve anti-bacterial action and enhanced wound healing efficacy in diabetic foot ulcer

**DOI:** 10.1016/j.reth.2024.03.006

**Published:** 2024-03-18

**Authors:** Lina Wang, Yan Yang, Weiwei Han, Hui Ding

**Affiliations:** a Department of Endocrinology, Qingdao Chengyang District People's Hospital, Qingdao, 266109, PR China; b Department of Dermatology, Qingdao Chengyang District People's Hospital, Qingdao, 266109, PR China; c Department of Medical Laboratory, Qingdao Huangdao District Central Hospital, 266555, PR China

**Keywords:** Diabetic foot ulcer, *Centella Asiatica* extract, Poloxamer, Anti-bacterial action, Cell proliferation, Wound healing

## Abstract

Diabetic wounds can occur as a prevalent complication among people diagnosed with diabetes, frequently resulting in the necessity for amputation. The cause and effect of diabetic foot ulcer is complex, involving multiple factors. In the present study, wound healing strategies utilizing nanomaterials have proven to be effective in battling bacterial infections and improve wound regeneration. Poloxamers (PLX) exhibit extensive potential as a viable option for the development of nanomedicines owing to their inherent characteristics of self-assembly and encapsulation. This study aims to design and develop a PLX/ZnO nanocomposite incorporated with *Centella Asiatica* extract (CAE) for the multi-functional action in the diabetic wound healing treatment. Subsequently physico-chemical characterizations, such as XRD, FTIR, and TEM observations, demonstrated that the ZnO were evenly distributed through the PLX framework. The developed nanocomposite was biocompatible with mouse fibroblast cell line (L929), and it had multiple beneficial characteristics, such as a rapid self-healing process and effective antibacterial action against G^+^ and G^−^ bacterial pathogens. After being treated with the developed formulation, skin fibroblast cell line and HUVECs demonstrated a substantial increase in their *in vitro* cell proliferation ability, migration, and tube-forming abilities. The utilization of a CAE@PLX/ZnO nanoformulation presents a viable strategy and a distinctive, encouraging composite for diabetic wound healing treatment.

## 1 Introduction

A primary risk factor for diabetes mellitus is impaired wound healing, which might have long-lasting negative impacts on quality of life, morbidity, and mortality. The current standard of care for diabetic wound treatment relies on early detection, preventative actions, and comprehensive instruction for patients [[1], [2], [3]]. Impaired wound healing in diabetes is triggered by an intricate aetiology, but the main factors include increased oxidative stress and chronic inflammation. Typically, wounds are vulnerable to bacterial infections that disrupt the healing process by prompting inflammation and instigating damage to the surrounding tissue. The process of wound healing is considered to be highly complex within the human body. An ideal therapeutic approach should possess the capability to accelerate wound healing, reduce microbial infections, maintain suitable microenvironments, and mitigate scar formation [4,5]. The field of wound healing has witnessed a growing number of novel therapies; however, certain treatments have proven to be ineffective in achieving favorable clinical outcomes. Wound re-epithelialization and fluid loss management are two examples of structural factors that contribute to these problems [6]. The influence that various treatment modalities have on the healing process of diabetic wounds, the part that underlying comorbidities play in holding back the healing process, and the identification of novel therapeutic targets for the purpose of enhancing wound healing in diabetic patients are all topics that will be discussed. We will be able to effectively address the gaps in the current research on diabetic wound healing and work toward developing interventions that are more targeted and effective for this patient population if we narrow our focus to these specific areas [7,8]. These considerations have prompted a new era in the use of nanomaterials for wound healing treatments to emerge from the realm of nanotechnology. This approach offers potential solutions for expediting the process of wound healing, while also providing unique properties as bactericidal agents [[4], [5], [6]]. With simple manipulation of material type, nanoparticle size, and surface charge, the biochemical properties of nanoparticles, such as hydrophobicity, connection with biologically active compounds, and tissue penetration at varying depths, may be easily tailored to different kinds of wounds [[7], [8], [9]]. Two primary approaches have been devised for the utilization of nanomaterials derived from both inorganic and organic sources, respectively. Nanoscale materials possessing antimicrobial properties are commonly incorporated into polymers, particularly biopolymers derived from abundant natural sources, or nanocarriers employed for encapsulating the active agent. Biomedical nanomaterials have garnered increased attention in recent times due to their notable biological attributes and their applications in the field of biomedicine. Nanoparticles of metal oxides, such as zinc oxide (ZnO), silver (Ag), and cerium oxide (CeO2), in addition to other materials including graphene and carbon nanotubes (CNT), have exceptionally promising potential in the biomedical field. One of the most important metal oxide nanoparticles, zinc oxide nanoparticles (ZnO NPs) have unique physical and chemical characteristics that make them potential in different biomedical areas [10,11]. Moreover, ZnO NPs exhibit remarkable antimicrobial properties, as well as exceptional UV-blocking capabilities. Zinc is widely recognized as an indispensable trace element that is found abundantly in various tissues of human body, such as the brain, muscles, bones, and skin. Zinc, being a fundamental constituent of multiple enzyme systems, actively participates in the metabolic processes of the human body. It assumes vital functions in the synthesis of proteins and nucleic acids, as well as in hematopoiesis and neurogenesis [12]. In comparison to other metal oxide nanoparticles, ZnO NPs possess relatively lower toxicity and cost-effective, making them highly suitable for various biomedical applications. Among these uses are anti-cancer medications, drug delivery systems, antibacterial agents, diabetes medications, anti-inflammatory agents, wound healing and bioimaging applications [[13], [14], [15]]. Investigated the possibility of different sized ZnO NPs penetrating injured and allergic surface in an animal model of dermatitis. The results of the experiments showed that larger-sized ZnO could only reach the upper layers of damaged and allergic skin, whereas nanosized ZnO particles could reach the deeper layers of the skin. The findings of this study indicate that ZnO nanoparticles (NPs) of smaller size exhibited significant efficacy in mitigating skin inflammation in models of atopic dermatitis (AD), when compared to larger-sized ZnO NPs. Because of its antidiabetic, antifungal, antioxidant, anti-inflammatory, and antibacterial efficiencies [[16], [17], [18], [19]].

Recent years, self-assembled block copolymers (BCPs) have been instrumental in the creation of cutting-edge nanoscale systems that have revolutionized medication delivery. PLX, alternatively referred to as pluronics, are a distinctive category of synthetic triblock copolymers. A core hydrophobic chain of poly (propylene oxide) (PPO) and two hydrophilic chains of poly (ethylene oxide) make up these copolymers (PEO). The synthesized PLX show great promise for use in a range of biomedical applications due to their chemical characteristics, which include thermo-reversible behavior and self-assembly that is temperature sensitive, as well as their physiochemical qualities and biocompatibility. When administered intravenously to human subjects, pluronic block copolymers have been shown to exhibit a level of safety that is satisfactory, as was previously reported. In the context of its application as a wound healing cleansing agent, the utilization of poloxamer 188, which is characterized by a substantial ethylene oxide content of 80% and a molecular weight of 8350 Da, has been identified as a potential material for wound healing applications. It demonstrates efficacy as a bactericidal agent, effectively eliminating bacteria without causing damage to tissue, and providing assistance in preventing infection without inducing any adverse effects that have been observed [20,21].

**Fig. 1:**
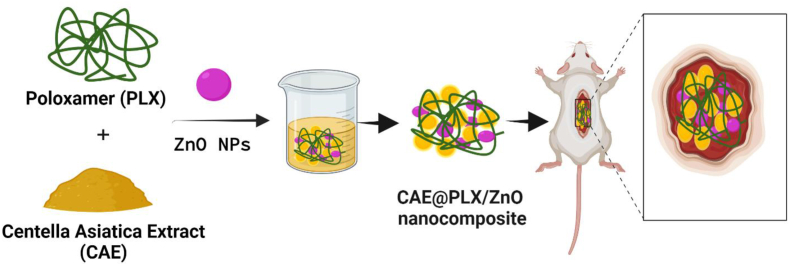
Schematic representation of the preparation of CAE-loaded PLX/ZnO nanocomposite for the treatment of diabetic foot ulcer.

The extracts from *Centella asiatica* (CAE) is widely utilized in traditional chinese medicine. Wound dressings formulated with CAE have an advantage in healing because it promotes collagen synthesis and fibroblast proliferation [[22], [23], [24]]. As a result, several biomaterials have been studied in relation to CAE's potential use as a wound dressing [[25], [26], [27]]. As a result, there is growing interest in using CAE in wound dressing applications, and a number of biomaterials have been studied [28,29]. The previous reports used incision and partial-thickness burn wound models in rats to examine the effects of consecutive CAE prepared with hexane, ethyl acetate, methanol, and water on wound healing. The positive attributes of wound dressings can be further augmented by incorporating natural bioactive agents. In order to achieve the goals of enhancing antibacterial activity and enhancing the effectiveness of wound healing in diabetic foot ulcer applications, the current investigation has designed and developed on the synthesis of PLX/ZnO nanocomposites that contain *Centella Asiatica* Extract (labelled as CAE@ PLX/ZnO). A highly effective and stable nanocomposite material with enhanced antimicrobial and wound healing properties is expected to be developed as a result of the combination of ZnO nanoparticles, CAE, and poloxamer, according to the hypothesis of the study. As a result of the incorporation of ZnO nanoparticles, it is anticipated that the nanocomposite material will possess powerful antimicrobial properties. Additionally, it is anticipated that the addition of CAE will contribute to the wound healing properties of the material. Poloxamer, which is well-known for its ability to stabilize and solubilize substances, is anticipated to improve the nanocomposite's overall performance in terms of stability. It is hypothesized that these three components will work together in a synergistic manner to produce a nanocomposite material that is both highly effective and stable, and that possesses enhanced antimicrobial and wound healing properties. The schematic representation (Fig. 1) displays that our developed nano-composited formulation showed great promise for effective chronic diabetic wound healing, which inhibited bacterial infection, reduced inflammation, accelerated fibroblast cell survival and proliferation, and facilitated improved wound healing.

## 2 Experimental section

### 2.1 Materials and methods

*Centella Asiatica* plant collected from region of eastern Shandong, PR China. Poloxamer (100%), Zinc nitrate (99.0%), Dichloromethane (99.80%), N-hexane, Ethyl acetate, Methanol, Ammonium hydroxide (28% NH_3_ in H_2_O, 99.99%) and Tween 20® were purchased from Sigma Aldrich in China.

### 2.2 Synthesis of *Centella Asiatica* extract (CAE)

The leaves of CAE were harvested from the local area and then dried in an oven at 50 °C before being milled into a powder. The CAE plant extract was prepared as following previously reported protocol with slight modification [30]. At room temperature, 1 kg of plant powder was macerated with 3–4 L of n-hexane for three days. The mixed filtrates were dried at low pressure until they attained a constant weight of 17.8 g, and then the n-hexane extract was produced (a yield of 0.18% for the fresh plant). After the marc was air-dried, it was macerated with ethyl acetate (32.7 g, 0.33% yield) and methanol (267.5 g, 2.68% yield), respectively, according to the procedure outlined above. Afterwards, 300 g of the dried marc was boiled in 2 L of distilled water for 3 h, strained, and dried to obtain 95.1 g of the hot aqueous extract (3.17% yield). Tween 20®, a 10% solution of polyoxyethylene (20) sorbitan monolaureate in distilled water, was used as a carrier to prepare 10% (w/v) of each extract for topical application. Ten grammes of powdered extract was mixed with 9 mL of vehicle. The vehicle was gradually added after the extract concentration reached 10%.

### 2.3 Preparation of zinc oxide nanoparticles (ZnO)

The production of ZnO was achieved using a precipitation method, as referenced [31]. To prepare dissolution, 50 mL of distilled water was used to make a solution that contained Zn(NO_3_)_2_ at a concentration of 0.05 M. The mixture was treated with ultrasonic waves for 15 min while being constantly mixed. The ultrasonic treatment was performed at a power of 100 W and a frequency of 30 kHz, being sure to keep the temperature at 21 ± 2 °C throughout. Gradually adding 50 mL of 0.1 M NH_4_OH dropwise until a white precipitate formed brought the solution's pH down to around 9. After a 30-min stirring period, the resulting solution was subjected to periodic washing using distilled water and methanol in order to eliminate residual components. Subsequently, the precipitation underwent modification and was subjected to a drying process at a temperature of 120 °C for duration of 2 h in a hot air oven.

### 2.4 Synthesis of CAE loaded onto PLX/ZnO nanocomposites

The method of ultrasonication was employed to fabricate nanocomposites consisting of CAE encapsulated on PLX/ZnO. The process involved the dissolution of CAE in dichloromethane, resulting in the formation of CAE loaded PLX. Subsequently, these loaded PLX nanoparticles were utilized in the fabrication of nanocomposites. Subsequently, a distinct concentration of ZnO solution at boiling point (approximately 100 °C) was introduced into the PLX solution containing CAE. This was carried out in a controlled environment using ultrasonic methods at a flow rate of 0.2 mL/min (specifically, an ultrasonic power of 100 W at a frequency of 30 kHz). The dichloromethane was evaporated more easily from the reaction mixture after 30 min of agitation and sonication. The ultimate outcome underwent ultra-centrifugation at a temperature of 4 °C for 10 min. The nanocomposites containing CAE loaded on PLX/ZnO matrix were subsequently subjected to a drying process. The advantages of these antibiotic nanoparticles can be readily and efficiently synthesized through cost-effective methodologies.

### 2.5 Characterization methods

The nanocomposites were subjected to the Fourier transform infrared (FTIR) spectra of the CAE, ZnO, and CAE loaded PLX/ZnO nanocomposites were acquired using a PerkinElmer FTIR spectrometer (Beaconsfield, Bucks, UK). The spectra were collected within the wavelength range of 4000 to 400 cm^−1^. The X-ray diffraction patterns of nanocomposites were examined using a Rigaku diffractometer equipped with Cu-Kα radiation. The measurements were conducted within a 2θ range of 10–80°, employing a voltage of 40 kV, a current of 40 mA, and a scan rate of 0.02 s^−1^. The nanocomposites of PLX and ZnO, as well as those including CAE, were studied using fluorescence spectrophotometers (F-4500; Hitachi, Tokyo, Japan). The thermal properties of the developed samples were assessed using a thermogravimetric analyzer (TGA), specifically the TGA Q50 T. The instrument used in this study was an Instrument-Water LLC (Newcastle, DE, USA). The scanning electron microscopy (SEM; JEOL 6460LV) technique was employed to observe morphological structure of the CAE loaded onto PLX/ZnO nanocomposites. The DSC thermograms of nanoparticles were obtained by employing a DSC 882e, Mettler Toledo instrument. The temperature range investigated ranged from 25 to 900 °C. The nanoparticles were subjected to a heating rate of 5 °C min and a constant nitrogen flow of 100 mL min^−1^. The TEM images of the synthesized nanocomposites were acquired using a TEM model FEI Technai G2 20S-TWIN (USA). In this experiment, the CAE loaded PLX/ZnO nanocomposites were dispersed in distilled water and subsequently applied onto a 3 mm copper grid. The retention factors were measured with a liquid chromatograph and an Elite LaChrom HPLC Merck-Hitachi (Merck, Darmstadt, Germany) with a DAD (Diode Array Detector L-2455) detector, pomp L-2130, and a manual sample injection valve equipped with a 20 μL loop and EZChrom Elite software system manager as a data processor. The column was a Zorbax Eclipse XDB-C18 (Agilent Technologies, Santa Clara, CA, USA); (150 mm × 4.6 mm I.D., 5 μm).

### 2.6 Antibacterial activity

The present study investigated the antibacterial efficacy of ZnO, PLX/ZnO, and CAE loaded PLX/ZnO nanocomposites against *Staphylococcus aureus (S. aureus), Streptococcus pneumonia (S. pneumonia), Pseudomonas aeruginosa (P. aeruginosa),* and *Escherichia coli (E. coli)* bacterial strains through the implementation of the well diffusion method. Petri plates were prepared by adding 25 mL of sterile Muller Hinton agar media. Subsequently, each bacterial pathogen was individually inoculated onto Muller Hinton agar media in separate plates using a swab. The experiment involved evaluating the antibacterial activity of selected samples dispersed in dimethyl sulphoxide at a concentration of 2 mg mL^−1^. The levels of the zone of inhibition were measured 24 h after incubation overnight at a temperature of 37 °C. In this study, the positive control utilized was the standard antibiotic amoxicillin at a concentration of 2 mg mL^−1^.

### 2.7 *In vitro* biocompatibility assay

The effect on the cytotoxicity of the prepared wound closure materials was determined using mouse fibroblast L929 cells as model cells. The study investigated the cell viabilities of ZnO, PLX/ZnO, and CAE loaded PLX/ZnO nanocomposites at various concentrations (ranging from 0 to 200 μg/mL) and incubation times (12, 24, 48, and 72 h). The cell counting kit-8 (CCK-8) method was utilized for the purpose of this research.

### 2.8 *In vitro* cell proliferation assay

The nanocomposited wound closure material was sterilized prior to performing any biological and animal tests. The prepared nanocomposites were initially dropped onto a 24-well plate. After that, a volume of 500 μL containing L929 cells (1 × 10^5^ mL^−1^) was introduced into the well plate and incubated for different incubation hours (0–96 h) and different incubation time (1, 3 and 5 days). Cellular growth and proliferation were seen using microscopic method. The cells within the nanocomposite that developed endured staining using AO/EB for morphological observations. This process was performed at a temperature of 37 °C and within an environment consisting of 5% CO_2_ and 95% air with humidity. The fluorescent microscopy technique (Olympus, Japan) was employed to observe the rates of cell proliferation, survival, and death.

### 2.9 *In vitro* wound scratch assay

Using L929 cells, an *in vitro* wound model was created to examine the possibility of nanocomposite groups in wound closure. The L929 fibroblast cells was cultured on a 48-well plate with a cell density of 1.2 × 10^5^ in full DMEM medium until about 90 % confluence was reached after 24 h. To induce starving circumstances for 24 h and agreement that only cell migration leads to wound closure, after 24 h the media was withdrawn and rinsed with PBS. Then, 0.5 mL of DMEM (supplemented with 0.1% FBS) was added. A sterile 200 μL micro-tip was used to make a small scratch at the base of each well, which was then washed twice with PBS to remove any debris. Then, cell lines were treated with prepared samples in each well, while DMEM with 10% FBS served as a control. The ratio of the wound area covered by migrating cells was then used to determine the closure of the wound at 0, 6, 12, 24, and 48 h. The area of cell migration at the specified duration was determined using ImageJ software compared to the area of cell proliferation at 0 h. The images were visualized by fluorescence microscopic method (Olympus, Japan) using AO staining.

### 2.10 *In vitro* tube formation assay

The HUVECs were cultured for 48 h with either PBS and prepared samples: control, ZnO, PLX, and CAE@PLX/ZnO. A solution of Matrigel (200 μL) from BD Biosciences, USA, was evenly distributed into 12-well plates and allowed to gel at 37 °C for some minutes. Afterwards, cells (2 × 10^5^ cells/well) were seeded onto polymerized Matrigel from various groups. To assess tube formation, an inverted microscope (Olympus, Japan) was employed after 10 h of incubation. Quantification of the results was done by measuring the number of tubes that formed in the corresponding image. The experiments were performed triplicate for each sample.

### 2.11 Analysis of collagen contraction assay

In a 2 mL volume of type I bovine collagen solution were treated L929 fibroblast cells with ZnO, PLX/ZnO, and CAE@LPX/ZnO samples for 8, 16, and 24 h, respectively as previously reported. Then, observed and recorded the contraction of each collagen gel at 8 and 24 h. We evaluated the percentages of contraction at different points with the control, which did not receive any treatment.

### 2.12 qRT-PCR for inflammatory cytokines

For three days, L929 cells were cultured in six-well plates. Using PBS, the cells were washed twice. Before treatment, the L929 fibroblast cells were transferred to serum-free DMEM for one day. The L929 cells that were cultured were exposed to LPS (1 μg/mL; Sigma-Aldrich, USA) for 4 h, followed by 30 min of preparation of samples. After that, we harvested the cells to isolate the total RNA and extract the proteins. Following the manufacturer's protocols, total RNA was extracted from cultured L929 cells using FavorPrepTM Tri-RNA reagent. 20 μL of diluted cDNA template, forward and reverse primers, 10 μL of SYBR Premix Ex Taq (Takara Bio, Inc.), and the Bio-Rad Real-Time PCR Detection System (USA) were utilized to perform real-time RT-PCR. For every sample, real-time PCR quantification was performed in triplicated and the average was determined. With GAPDH values as a reference, real-time PCR was used to examine expression levels.

### 2.13 Statistical analysis

The data are reported quantitatively as an average ± S.E. of 3–5 samples per group, and all the tests were performed in triplicate (n = 3). Using Tukey's test of ANOVA, we compared each group to the control. Significant results were indicated by a p-value<0.05, ∗∗p < 0.01, and ∗∗∗p < 0.001.

## 3 Result and discussion

**Fig. 2:**
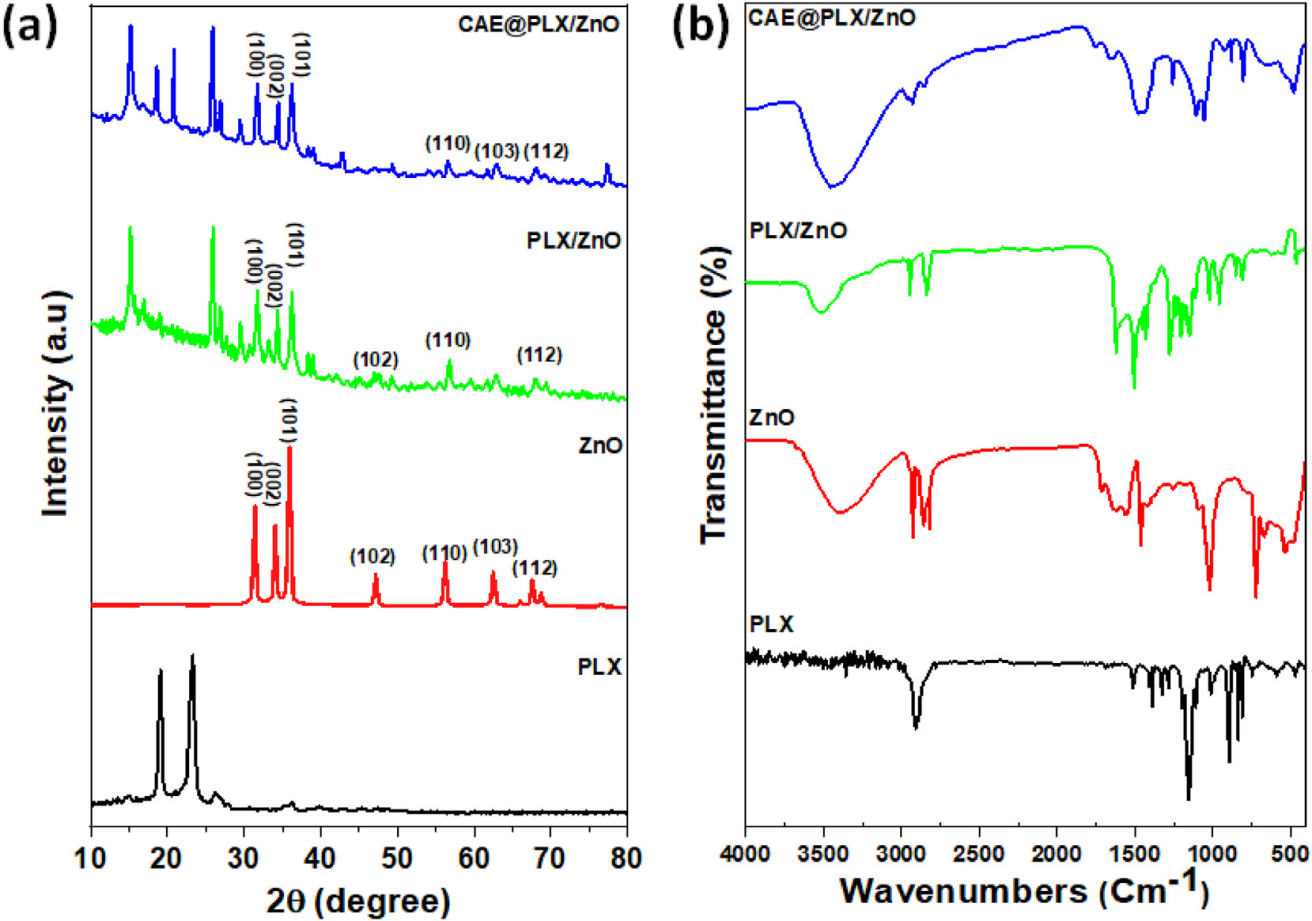
Nano-crystallinity and phase purity of the prepared formulations (PLX, ZnO, PLZ/ZnO, and CAE@PLX/ZnO) were analyzed by XRD (a), and structural interactions and chemical structure were determined by FTIR (b).

The X-ray diffraction pattern of the bare samples, which include Poloxamer and ZnO, as well as the composite materials in their prepared formulation is displayed in Fig. 2 (a). X-ray diffraction (XRD) data revealed that the nanocomposites, which included PLX, ZnO, PLX/ZnO, and CAE loaded PLX/ZnO nanocomposites, possessed a well-defined crystalline structure that was clearly observed. The peak positions that were obtained at 2θ positions 31.4, 34.2, 36, 47, 56.36, 62.64, 66.15, 67.65, and 68.9 are associated with the lattice plane of (100), (002), (101), (102), (110), (103), and (112), respectively. These peak positions provide evidence that the ZnO particles possess a crystalline structure. The lattice planes that were obtained demonstrated that the ZnO particle possesses a hexagonal wurtzite structure. Furthermore, the observed XRD data that has been strong correlated with the JCPDS card number 36–1451. The crystalline nature of the PLX matrix is shown by the position of the distinctive peaks, which are at 19.1 and 23.2, respectively. In addition to this, the positions of the peaks were slightly moved, and peaks disappeared in the composite system, which showed that the ZnO and CAE successfully encapsulated into PLX matrix network.

The FTIR spectral analysis is an important method that can identify which substances interact with one another. Based on the results of the FTIR observation, the interaction between the CAE and ZnO, together with the complexation of PLXs, was observed. As shown in Fig. 2 (b), ATR-FTIR spectra established that CAE@PLX/ZnO composites that have been successfully formed with the presence of ZnO, PLX and CAE components. There was a characteristic peak at 722 and 669 cm^−1^, which corresponded to the Zn–O stretching mode of vibrations [32]. In addition, the spectra of the PLX polymer revealed typical peaks at 3450, 2888, and 1115 cm^−1^. These peaks correspond to the stretching vibrations of hydroxyl and carbonyl link forms, which are characterized by the O–H, C–H, and C–O stretching vibrations. The result of the spectral observation displayed that some band locations of the compounds altered and that some of the peaks were disappeared, it was determined that the sample had formed a complex. As a result, the successful interaction of the functional groups is demonstrated by the shifts in the CAE and ZnO complex as well as the appearance of new peaks. During the process of incorporating CAE and ZnO into the PLX polymer matrix, the interaction became considerably more effective, as evidenced by the greater peak intensity of the major peaks presented in the prepared nanocomposites.

**Fig. 3:**
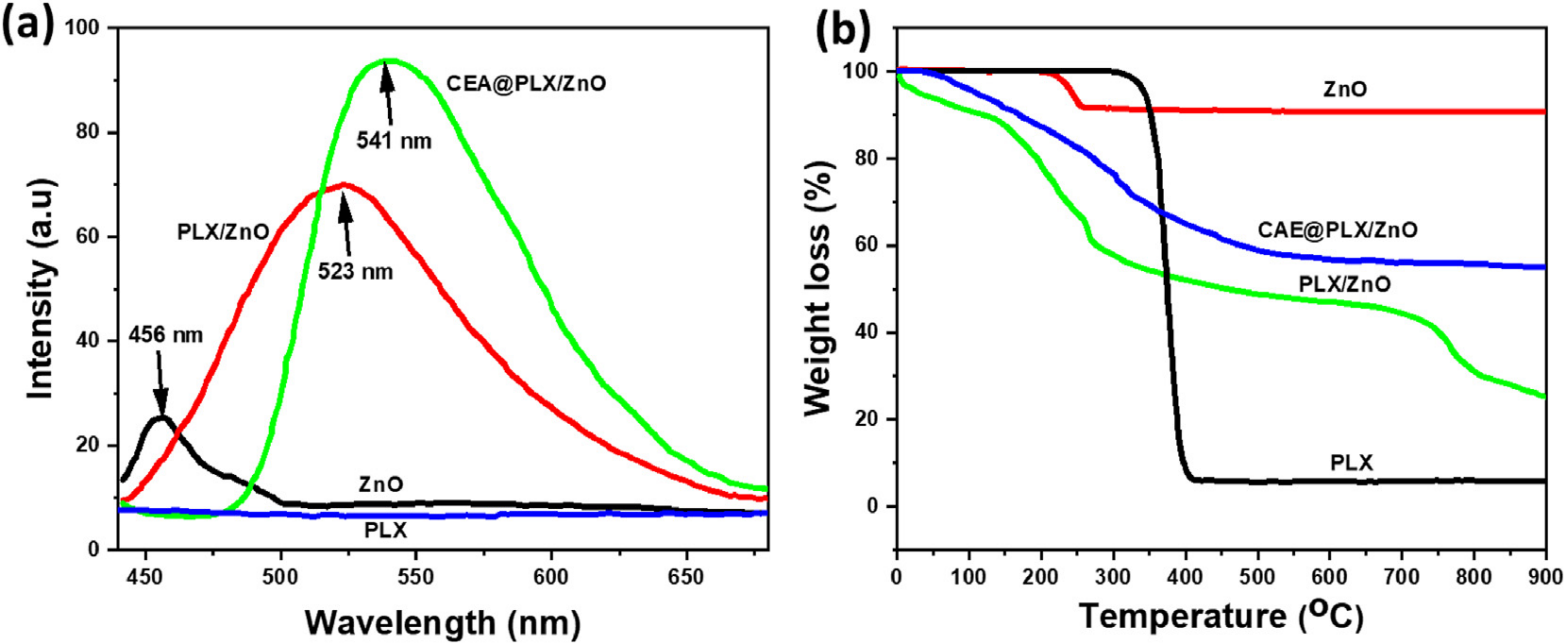
The fluorescence properties and thermal stability of the prepared materials (PLX, ZnO, PLZ/ZnO, and CAE@PLX/ZnO) were examined by fluorescence spectra (a) and TGA (b).

Fig. 3 (a) displays the fluorescence spectra of the PLXs as well as the prepared ZnO, PLX/ZnO, and CAE loaded PLX/ZnO nanocomposites samples. These spectra were obtained at an excitation wavelength of 541 nm. When measured at a wavelength of 456 nm, the ZnO nanoparticle displayed the excitation peak position. After careful observation, it was found that the chemical mixture consisting of PLX and ZnO exhibited a wavelength of around 523 nm, which is somewhat different from the ZnO's accurate excitation wavelength. While this was going on, the intensity of the peak position increased significantly when compared to the samples that included bare ZnO and PLX. On the other hand, when the combination was combined with the PLX polymer, the excitation wavelength grew even further, reaching 541 nm due to the higher excitation wavelength, and the peak intensity even increased. Therefore, the presence of the drug CAE, ZnO, and the PLX matrix was validated by the enhanced peak intensity of the samples as well as the shift in the excitation wavelength.

Using thermogravimetric analysis (TGA), we compared the as-prepared and unprocessed materials. Fig. 3 (b) displays the TGA profiles of the samples. The remarkable weight loss percentage observed at 350 °C for PLX polymer is in line with its high heat stability. A weight reduction of about 10% in the ZnO particles was indicative of the elimination of the water molecule (moisture) or the –OH functional group. The presence of the excellent stability of metal oxide nanoparticles, the ZnO nanoparticle exhibited good thermal stability over the investigated range. Even though it only shows a 40% weight reduction at 300 °C, the PLX/ZnO combination demonstrated an enhanced percentage of weight loss. The addition of the ZnO nanoparticles is responsible for the composite's enhanced thermal stability. At roughly 750 °C, a third weight loss was observed, which meant that the ZnO in the composite had completely decomposed. The prepared nanocomposites demonstrated much better thermal stability compared to those without PLX. Due to the PLX polymer's hydrophobic properties, the composite sample exhibited no signs of moisture. Weight loss for the composite begins at approximately 120 °C, primarily as a result of the polymer composition, and continues gradually up to 400 °C. Additionally, similar to the CAE and ZnO samples, this one exhibited excellent stability with no material loss. The composite sample's exceptional thermal durability allows it to be utilized in real-world applications at temperatures as high as 400 °C.

**Fig. 4:**
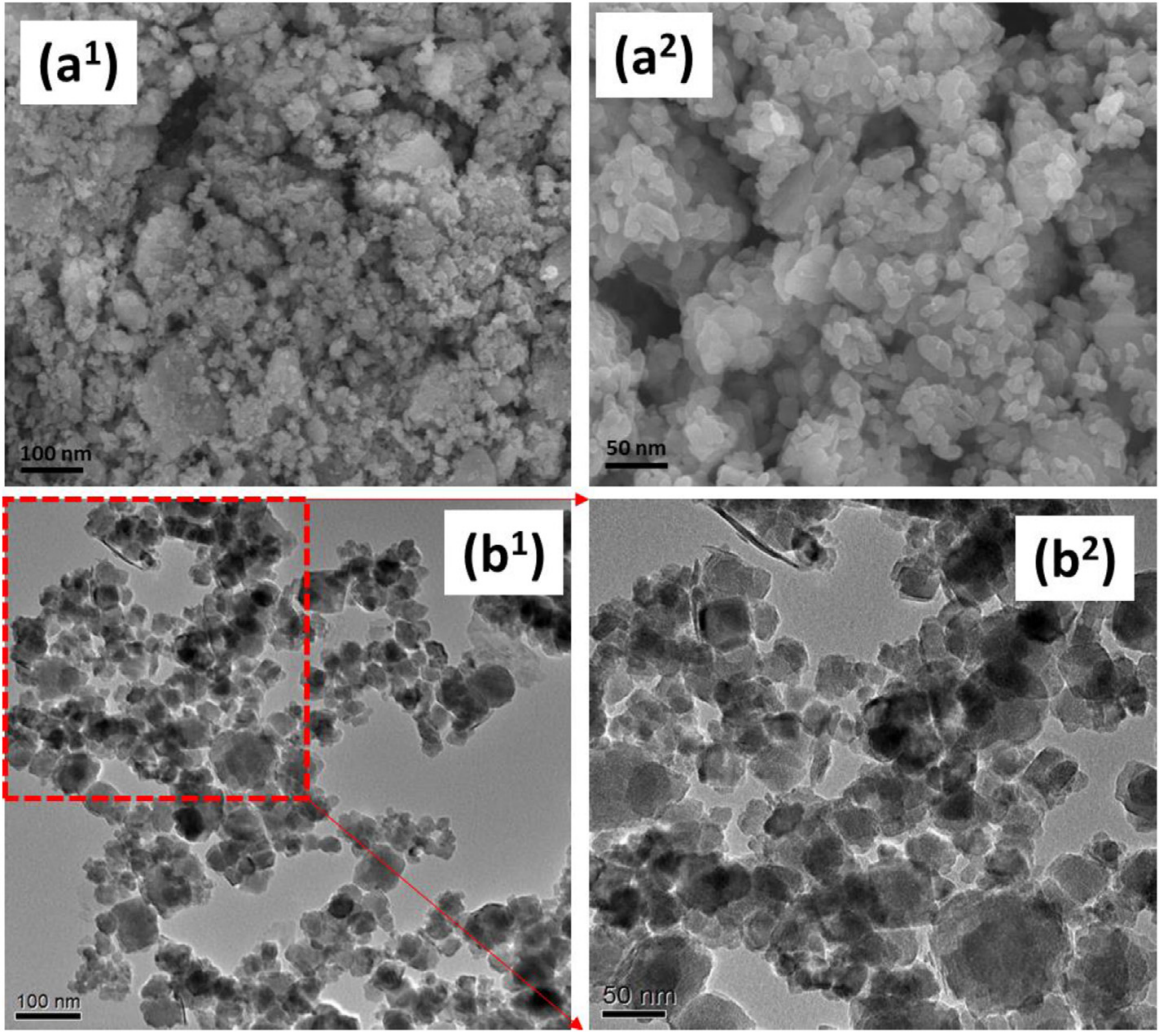
The morphological structure and distribution of nanoparticles in the prepared CAE@PLX/ZnO nanocomposite was observed by SEM (a^1^ & a^2^) and TEM (b^1^ & b^2^) analyses. Scale bar 100 nm & 50 nm.

The morphological structure and shape of the prepared nanocomposites of CAE@PLX/ZnO were characterized by SEM analysis as exhibited in Fig. 4 (a1 & a2). According to these measurements, the surface is uniformly covered with spherical nanoparticles that are all about the same size, which is less than 100 nm. It is plausible that the fabrication strategy in the nanocomposites of CAE@PLX/ZnO is responsible for the observed morphologies. Fig. 4 (b1 & b2) displays that TEM observation of CAE@PLX and ZnO NPs to examine particles size and distributions. The atomic configurations visible in great detail through the use of the TEM's high resolution can explain the nano particles dispersion and uniform spherical shape, which has an average diameter of approximately 50 nm. In addition, HPLC data reveals that the before and after encapsulation of centella asiatica extract-loaded poloxamer/ZnO, which is further evidence of the nanocomposites formation and the data shown in Figure S1.

**Fig. 5:**
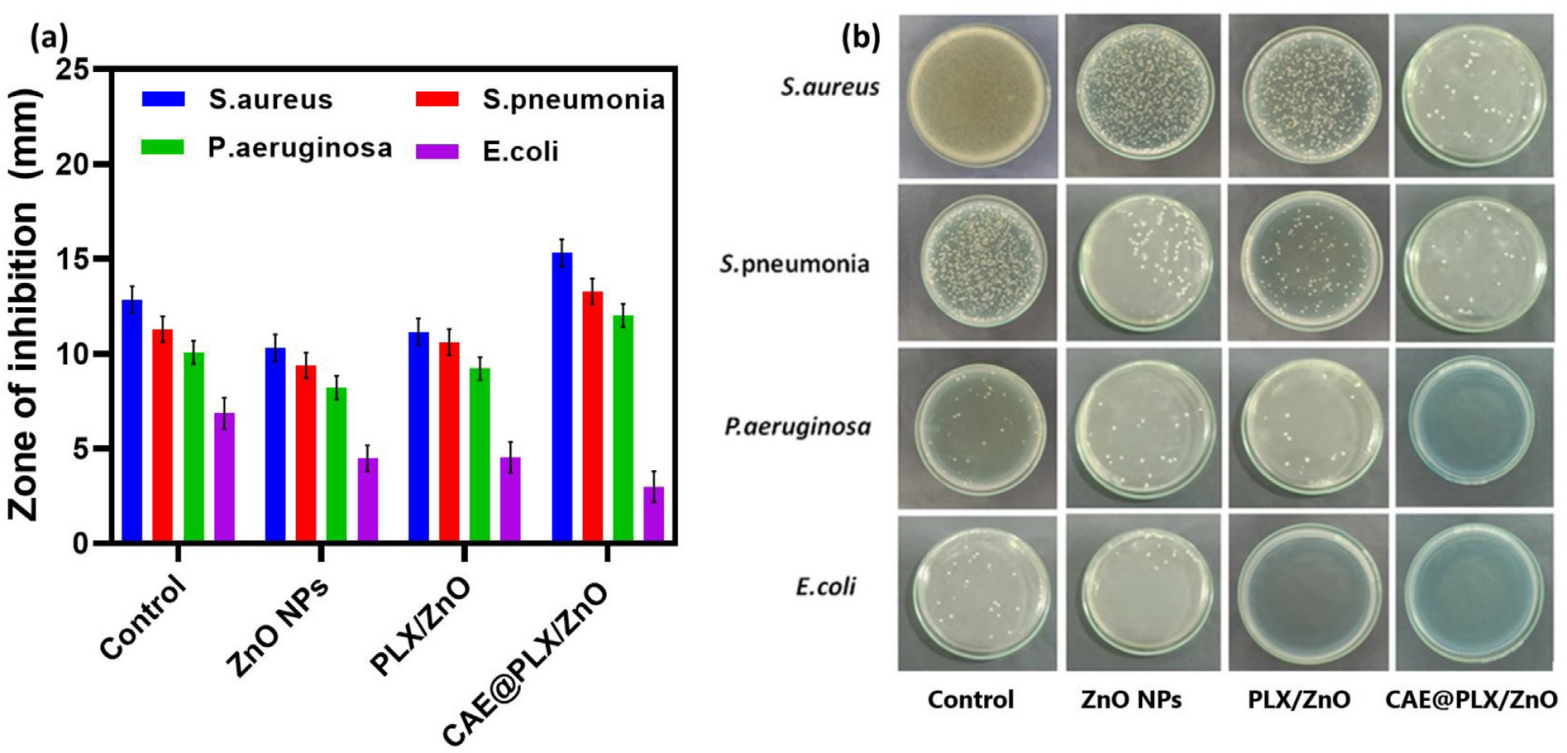
a) Zone of inhibition assay and (b) MBC photographs of control, PLX, ZnO, PLZ/ZnO, and CAE@PLX/ZnO against bacterial pathogens (*E. coli, P. aeruginosa, S. pneumonia, and S. aureus*).

We used the basic petri dish diffusion (zone of inhibition) method and colony forming unit (CFU) method to examine the antibacterial activity of the PLX, ZnO, PLX/ZnO, and CAE@PLX/ZnO with *Staphylococcus aureus (S. aureus)*, *Streptococcus pneumonia (S. pneumonia)*, *Pseudomonas aeruginosa (P. aeruginosa)*, and *Escherichia coli (E. coli)* bacterial pathogens. Antibacterial materials are in great demand because bacterial infections are a leading cause of wound healing delays in clinical practice. The developed hydrogels were examined for their antibacterial behavior using different bacterial pathogens in the present study. As shown in Fig. 5a, the zone of inhibition increased, whereas CAE@PLX/ZnO showed the highest antibacterial activity among the prepared nanomaterial formulations. The zone inhibition was ∼11 mm for the ZnO sample, ∼13 mm for the PLX/ZnO sample, and ∼17 mm for the CAE@PLX/ZnO nanocomposite. Importantly, the ZnO, PLX/ZnO, and CAE@PLX/ZnO nanocomposite groups demonstrated a substantial decrease in bacterial colony counts when compared to the control treatment group (Fig. 5b). The essential characteristics of CAE had been believed to be responsible for the nanocomposite's superior antibacterial activity.

**Fig. 6:**
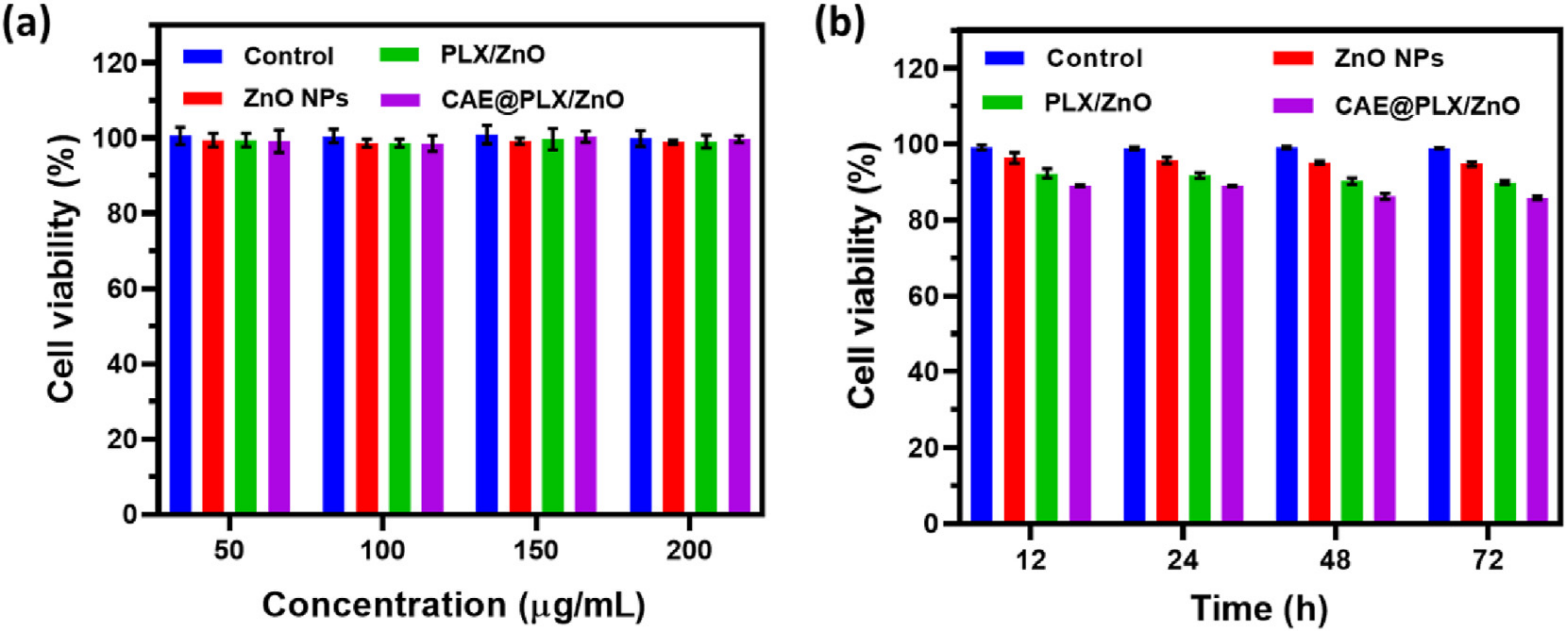
*In vitro* cell viability of L929 cells treated with prepared formulations (control, PLX, ZnO, PLZ/ZnO, and CAE@PLX/ZnO nanocomposite) with (a) different concentrations (50–200 μg/mL) and (b) different incubation periods (0–72 h).

**Fig. 7:**
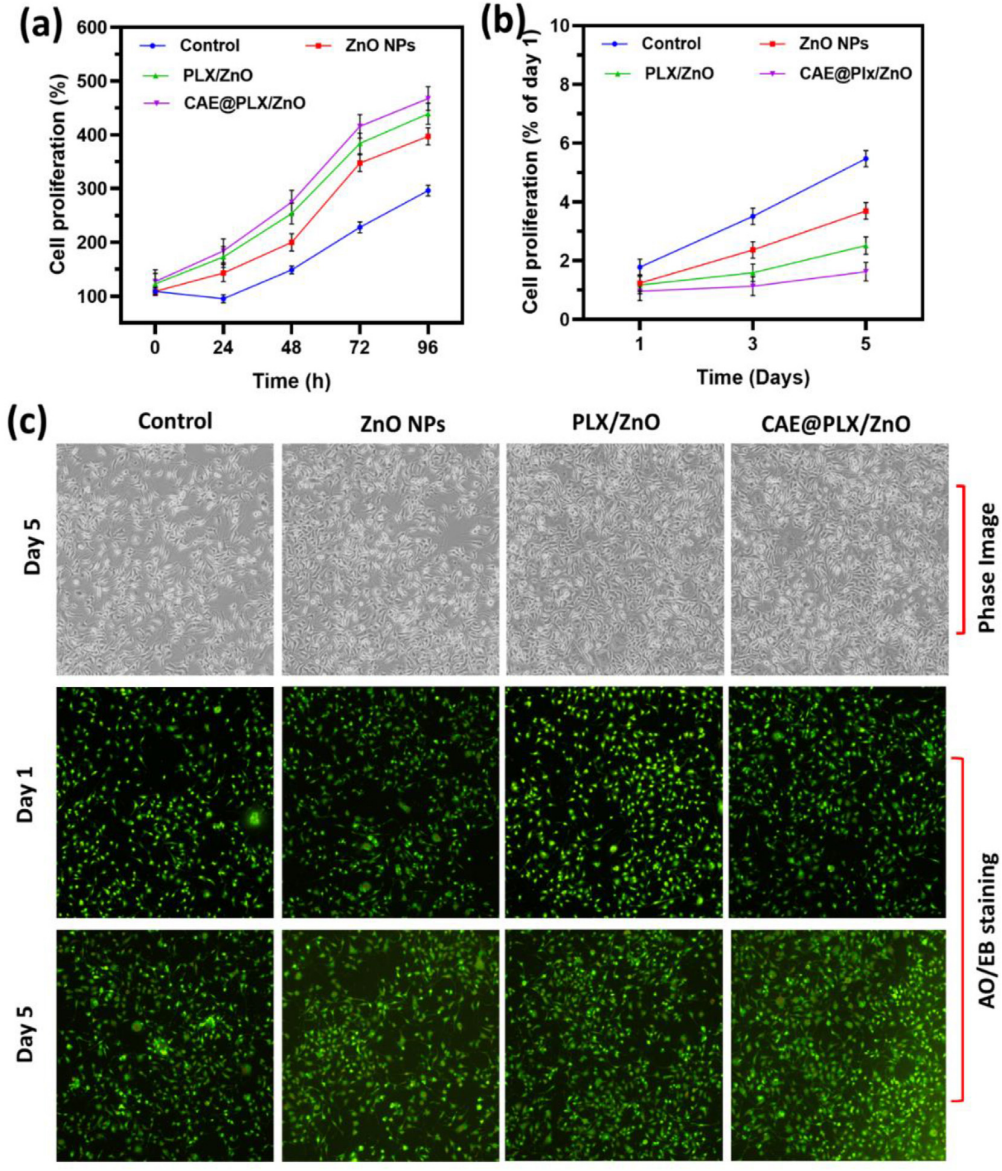
*In vitro* quantitative live cell number (a) and cell proliferation (b) observations of prepared nanocomposite formulations in different incubation periods; (c) *In vitro* qualitative observations of L929 cells treated with different nanocomposite formulations under phase contrast microscopic method and Fluorescence microscopic method (AO/EB staining).

The cell compatibility and survival rate of the prepared nanoformulations was examined by utilizing L929 cells as shown in Fig. 6a. We changed the concentrations of the positive control, ZnO, PLX/ZnO, and CAE@PLX/ZnO up to 200 μg/mL. The prepared CAE loaded nanocomposite showed greater cell compatibility with less toxicity effect. Meanwhile, the ZnO nanoparticles and ZnO/PLX materials indicated that slight variation in cytotoxicity across all doses. The change in cell viability is monitored every 12 h. In contrast, the manufactured ZnO nanoparticles, PLX/ZnO and CAE@PLX/ZnO nanocomposites showed excellent cell interaction and maintained 90% viability even after 24 h. Furthermore, the cell viability reaches a controlled 90% level after 72 h Fig. 6b. After one day in culture, the cell proliferation experiment (Fig. 7a and b) revealed that all of the tested materials maintained a comparable degree of proliferation for L929 fibroblast cells. Nanocomposite administration with CAE enhanced cell proliferation compared to treatment with PLX/ZnO or ZnO nanoparticles. In addition, the CAE@PLX/ZnO nanocomposite showed a much greater effect on cell proliferation than the PLX/ZnO NPs in the absence of CAE. A 3-days culture on PLX/ZnO or CAE@PLX/ZnO nanocomposite resulted in an increase in cell proliferation of L929 cells. In many instances, cell adhesion and proliferation were slightly superior in CAE@PLX/ZnO nanocomposite compared to the PLX/ZnO composite. The fibroblasts had proliferated to a confluent stage in all of the culture wells in just 5 days, a result due to their rapid proliferation ability. Nanocomposite biocompatibility is an essential factor for their application in biomedical fields. To determine the nanocomposite's cytocompatibility, researchers entrapped L929 cells and employed live/dead staining to assess cell proliferation ability. The CAE@PLX/ZnO nanocomposites demonstrated an increase in the number of cells encapsulated by day 5 compared to day 1 (Fig. 7c), indicating that the PLX/ZnO and CAE@PLX/ZnO nanocomposites had excellent cell growth and proliferation facilitation.

**Fig. 8:**
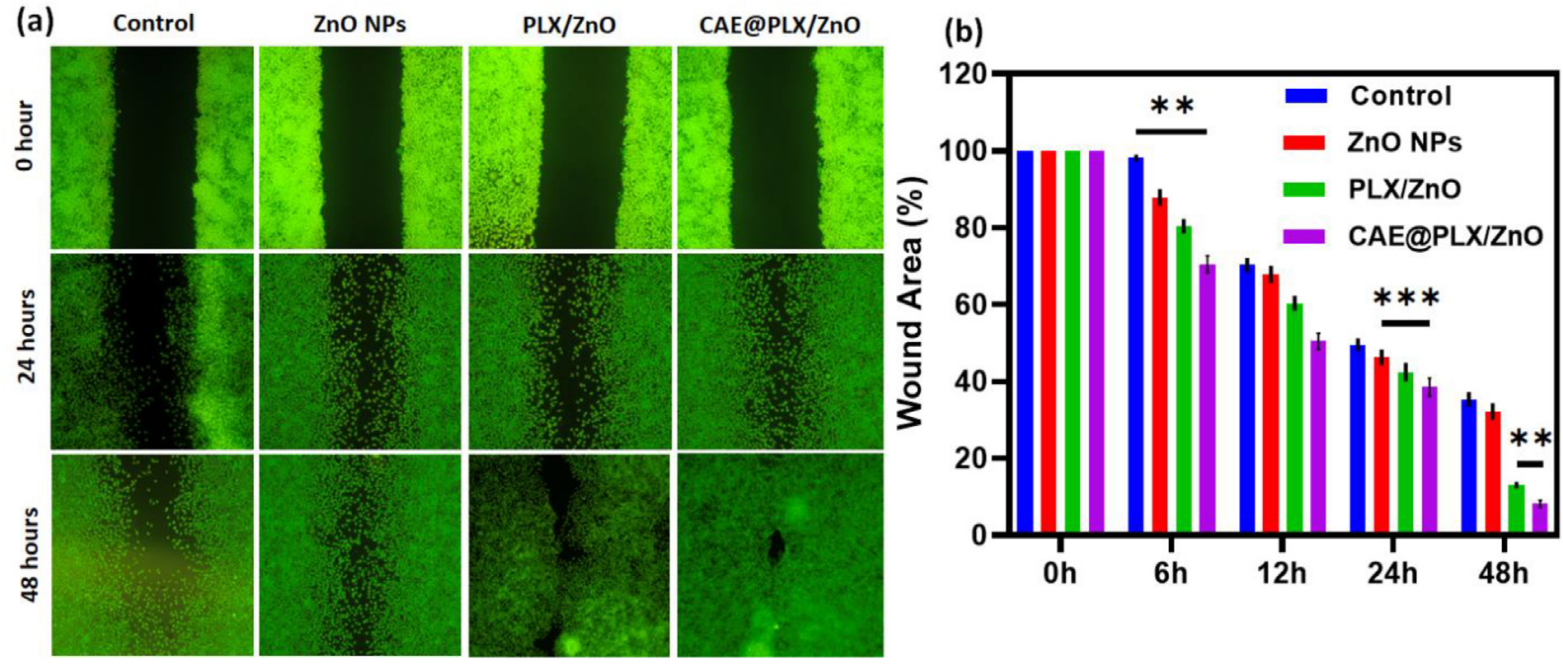
*In vitro* wound healing efficiency of prepared nanocomposite formulations was determined by wound scratch method using L929 cell line; (a) Fluorescence microscopic (AO staining) visualization of L929 fibroblast cell treated with Control, PLX, ZnO, PLZ/ZnO and CAE@PLX/ZnO for 0, 24 and 48 h; (b) Percentage of wound area (%) in different time (0–48 h).

A successful wound closure depends on the migration of cells to the wound site. Previous studies have demonstrated that wound healing material, which promotes cells quickly in a lab setting, may significantly enhance wound healing. An *in vitro* wound healing model has been developed using skin fibroblasts (L929) cells. As exhibited in Fig. 8a, we can observe that L929 cell migration to fill the scratched region was used to evaluate the wound closure propensity of the treated groups. The treated groups moved their cells to fill the gap faster after 48 h compared to the control group (Fig. 8b). The results indicate migratory delays were observed in fibroblast (L929) cells treated with ZnO and PLX/ZnO nanocomposites. The CAE@PLX/ZnO treated group demonstrated a significantly greater and more beneficial effect on cell migration, with values of around 85% ±4.73 (p < 0.001) and 95% ± 5.37 (p < 0.01), respectively. Images taken under a microscope reveal that the results of the experiment suggest that the incorporation of nano-formulations into the CAE@PLX/ZnO nanocomposites enhanced cell migration and proliferation, which could lead to accelerated wound healing.

**Fig. 9:**
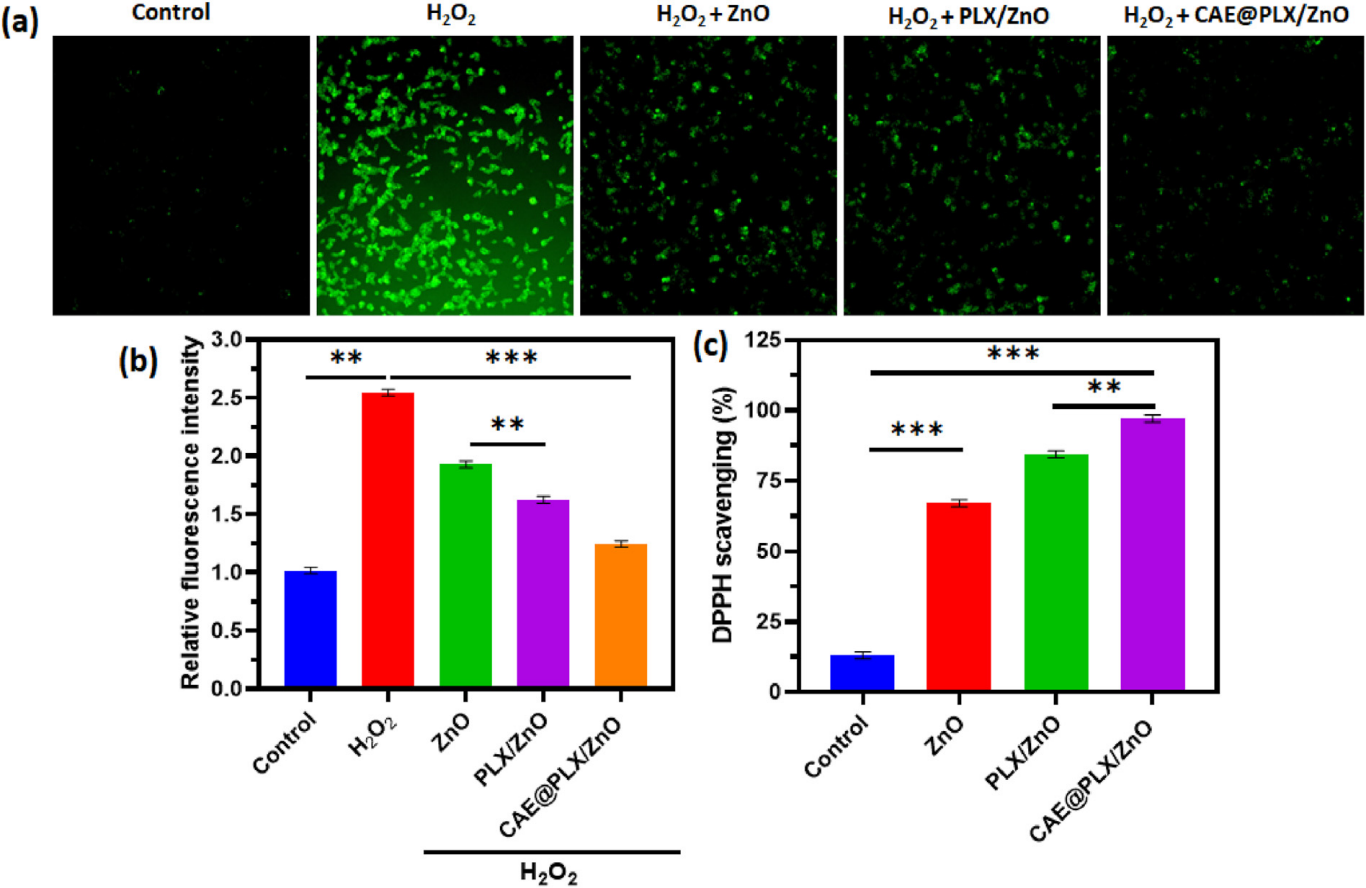
(a) *In vitro* ROS generation assay by H_2_O_2_ method under L929 cells treated with ZnO, PLX/ZnO, and CAE@PLX/ZnO nanocomposite using DCFH-DA staining under fluorescence microscopic method; (b) Relative fluorescence intensity and (c) Anti-oxidant ability of prepared nanocomposite formulation through DPPH assay.

In addition, a ROS-specific probe was employed to assess the intracellular ROS scavenging capability of the CAE@PLX/ZnO nanocomposite. After incubation with ZnO, PLX/ZnO, or CAE@PLX/ZnO, L929 fibroblasts were exposed to H_2_O_2_ stimulation to induce intracellular oxidative stress. ROS levels were then measured using DCFH-DA fluorescence in green. After being treated with H_2_O_2_, the green fluorescence intensity was 2.4 times higher than the PBS control as exhibited in Fig. 9a and b. A substantial reduction in fluorescence intensity was observed after incubating the cells with PLX/ZnO comprising CAE, suggesting that CAE@PLX/ZnO effectively mitigated intracellular oxidative stress. The capacity of CA extract to alleviate excessive oxidative stress in the diabetic wound microenvironment was demonstrated, in particular, when the intracellular ROS level was found to be normal after treatment with CAE@PLX/ZnO nanocomposite in its presence. Furthermore, as illustrated in Fig. 9 (c), the anti-oxidant ability of the developed nanocomposite was determined using a DPPH assay. The remarkable antioxidant activity of CAE@PLX/ZnO has been shown when DPPH levels were gradually decreased and DPPH scavenging activity was substantial when treated with PLX/ZnO nanocomposite containing CAE extract.

**Fig. 10:**
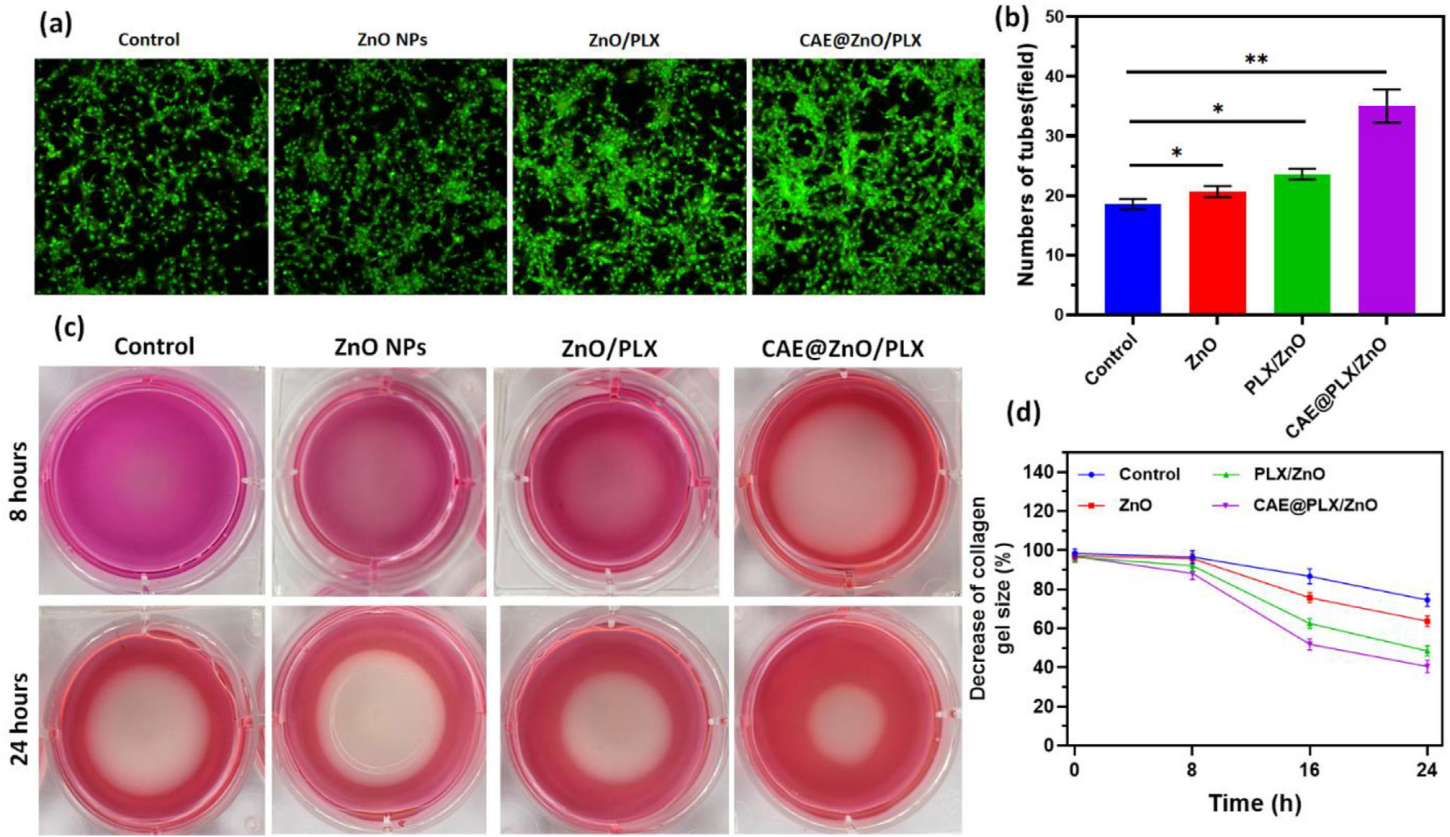
(a & b) *In vitro* tube formation analysis of prepared nanoformulation on HUVECs cells and (c & d) collagen contractility study by different treatment groups.

The purpose of this study was to examine the potential of as-prepared ZnO, PLX/ZnO, and CAE@PLX/ZnO samples to the migratory and tube-forming abilities of HUVECs after 48 h. Fig. 10a demonstrates the increased migratory capacity and Fig. 10b shows the increased tube-forming ability of HUVECs treated with PLX/ZnO and CAE@PLX/ZnO nanocomposites, respectively, compared to the control groups. The results showed that CAE@PLX/ZnO can increase the ability of HUVECs to proliferate, migrate, and form tubes, which can induce angiogenesis *in vitro*. Furthermore, a 3D type I collagen contraction assay was carried out to determine the effect of regulating the contractile capacity of L929 cells. Throughout a 24-h culture period, the collagen gels' sizes were measured at various time points (Fig. 10(c and d)). The collagen gel contraction was facilitated by the 8, 16 and 24 h treatment of L929 cells with prepared CAE@PLX/ZnO. This reduction was confirmed by both macroscopical observations and measurements of the gel size (Fig. 10d). Collagen gel contraction was significantly facilitated by subjecting L929 cells to CAE@PLX/ZnO for 24 h. The fibroblast L929 cells could have incorporated CAE with structured composites, allowing the gel to contract effectively.

**Fig. 11:**
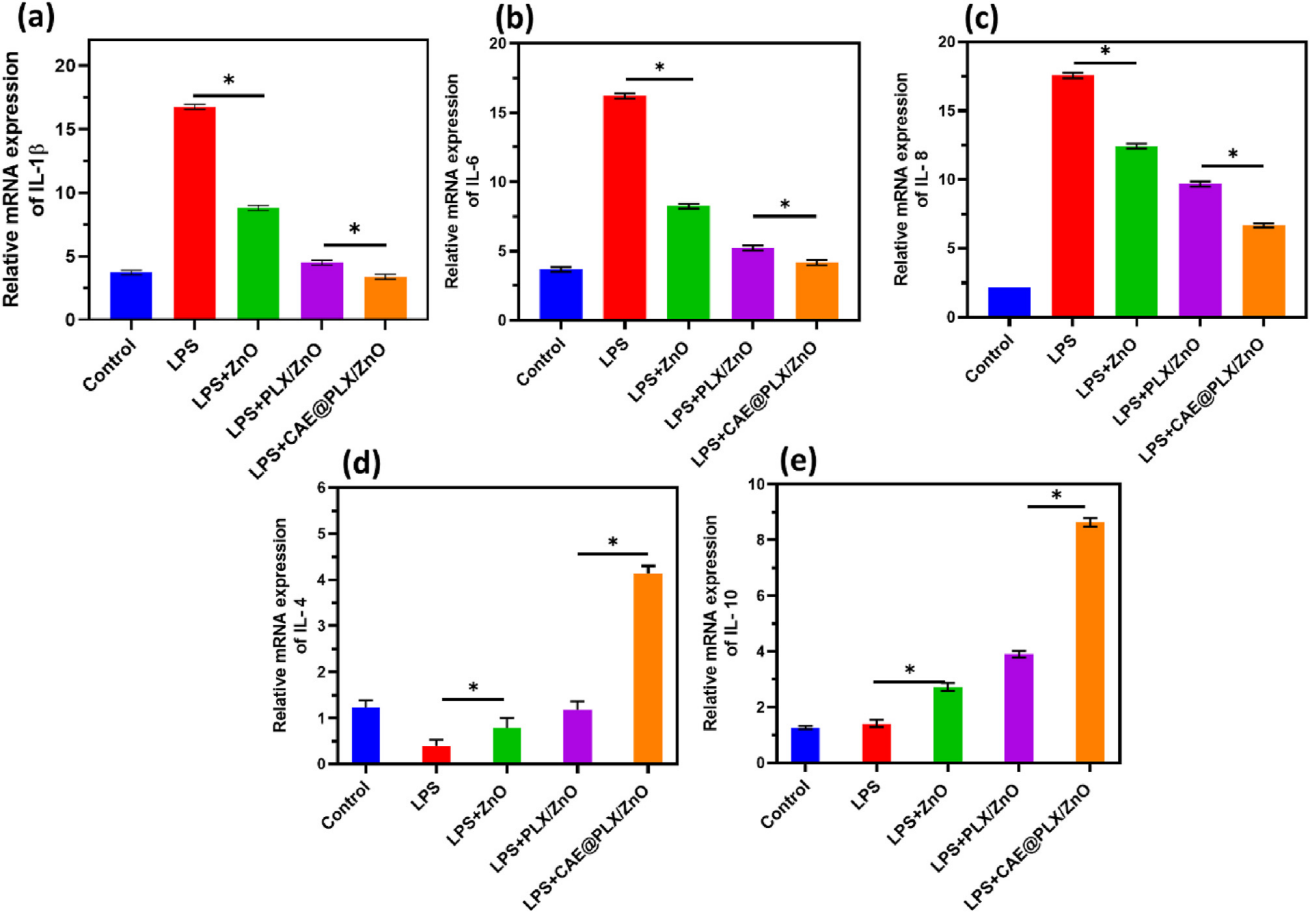
Real-time qRT-PCR analysis of pro-inflammatory cytokines ((IL-1β (a), IL-6 (b) & IL-8 (c)) and anti-inflammatory (IL-4 (d) & IL-10 (e)) cytokines in L929 fibroblast cells by treatment of LPS and prepared samples (control, ZnO, PLX/ZnO and CAE@PLX/ZnO).

After inducing inflammation in L929 cells with LPS, cells were cultured in the presence of prepared samples to see if CAE loaded PLX/ZnO nanocomposited formulations might decrease inflammatory responses. To investigate how CAE@PLX/ZnO treatment influenced the mRNA expression of pro-inflammatory (IL-1β, IL-6 and IL-8) and anti-inflammatory (IL-4 and IL-10) cytokines, RT-PCR was employed. Fig. 11(a and b & c) demonstrates that when L929 cells were treated with CAE@PLX/ZnO, the mRNA expression of IL-1β, IL-6, and IL-8, which were induced by LPS treatment, was significantly reduced. Additionally, following CAE@PLX/ZnO treatment, there was a notable increase in the mRNA expression of anti-inflammatory cytokines (IL-4 and IL-10), illustrated in Fig. 11d and e. Pro-inflammatory cytokines and chemokines, like IL-6 and IL-8, are released into the bloodstream when LPS causes inflammation. Data suggests that IL-6 and IL-8 play an important role in wound healing by triggering chronic inflammation. The anti-inflammatory cytokines, such as IL-4 and IL-10, suppress the immune response and prevent the production of cytokines that promote inflammation. These findings prompted us to believe that CAE@PLX/ZnO might suppress pro-inflammatory cytokines in L929 cells, which would indicate that it has an anti-inflammatory function. The outcomes of *in vitro* anti-bacterial investigations and cell treatment studies demonstrated that CAE@PLX/ZnO nanocomposited formulation exhibited greater cell proliferation, migration ability with effective potential of anti-oxidant and bactericidal inhibition ability, which leading to enhanced skin regeneration ability and therapeutic potential for diabetic wound healing.

They investigated the effectiveness of a nanocomposite that was very similar to this one in inhibiting the growth of bacteria in a study that was conducted [32]. They discovered that the nanocomposite exhibited powerful antibacterial activity against Gram-positive as well as Gram-negative bacteria, which is in line with the findings that we obtained. When it comes to the effectiveness of our nanocomposite against bacteria, this demonstrates that it is consistent with the research that has been done previously [33]. In addition, performed research that investigated the potential of a nanocomposite in the context of applications related to wound healing [34]. According to their findings, the nanocomposite facilitated a more rapid closure of wounds and also enhanced tissue regeneration, indicating that it has the potential to be utilized in biomedical applications [35]. In addition, our research demonstrates that our nanocomposite has the potential to be used in wound healing, which further substantiates its uniqueness and effectiveness in this particular application. Moreover, demonstrated a review in which they discussed the application of nanocomposites in the process of environmental remediation. They brought attention to the potential of nanocomposites in the removal of pollutants from water and soil, which is in line with the findings of our research on the effectiveness of our nanocomposite in the removal of heavy metals from water [36]. The novelty and effectiveness of our nanocomposite in environmental remediation applications is further highlighted by this comparative analysis with the existing body of literature. In general, the comparative analysis with the existing literature provides evidence that our nanocomposite is both effective and novel in a variety of applications [37]. These applications include antibacterial activity, wound healing, and environmental remediation.

## 4 Conclusion

In summary, we have developed the eco-friendly, cost-effective, non-toxic PLX/ZnO composited material encapsulated with CAE for effective regeneration of diabetic foot ulcer. Based on nanomaterials characterizations, we revealed that the hexagonal wurtzite ZnO (<11 nm) and CAE extract have been successfully encapsulated in the PLX nanocomposited network. The *in vitro* antibacterial studies exhibited that prepared CAE@PLX/ZnO nanocomposites have providing increased and efficient bactericidal inhibition against G^+^ and G^−^ bacterial pathogens. The developed wound closure nanocomposite material demonstrated for superior cell compatibility, cell proliferation, migration ability and angiogenesis capability on L929 and HUVEC cell lines. In addition, the developed material has been demonstrated for enhanced anti-oxidant anti-inflammatory action, which confirms that it would be potential candidate for diabetic wound healing application. For the following stage, in *in vivo* experiments involving animal models including diabetic wound infliction, we will be focusing on developed nanocomposites with enhanced healing potential that have suitable anti-infection characteristics. As part of this process, we will determine a therapeutic window for repairing wounds and ensure their safety to facilitate long-term wound healing for clinical purposes.

## Ethical statement

None.

## Funding

None.

## Data availability statement

Data will be made available on request to corresponding author.

## Author contributions

Lina Wang - Work design and conception.

Yan Yang - Synthesis, Characterization, Molecular and Biochemical analysis.

Weiwei Han - Formal analysis, Data curation and Validation.

Hui Ding - Helped-Supervised the research.

## Declaration of competing interest

The author states that none of the work presented in this study may have been influenced by any known conflicting financial interests or personal ties.
