# Supplementary material for: Novel design and development of Centella Asiatica extract - loaded poloxamer/ZnO nanocomposite wound closure material to improve anti-bacterial action and enhanced wound healing efficacy in diabetic foot ulcer
Source: Regen Ther. 2024 Mar 18;27:92–103. doi: 10.1016/j.reth.2024.03.006 (PMC10963185; doi:10.1016/j.reth.2024.03.006)
Supplement: Multimedia component 1 [file mmc1.docx]

**Novel Design and Development of Centella Asiatica Extract -loaded poloxamer/ZnO nanocomposite wound closure material to improve anti-bacterial action and enhanced wound healing efficacy in Diabetic foot ulcer**

**Lina Wang^1,#^, Yan Yang^2,#^, Weiwei Han^3^, Hui Ding^3,*^**

^1^Department of Endocrinology, Qingdao Chengyang District People's Hospital, Qingdao -266109, PR China

^2^Department of Dermatology, Qingdao Chengyang District People's Hospital, Qingdao - 266109, PR China

^3^Department of Medical Laboratory, Qingdao Huangdao District Central Hospital - 266555, PR China.

^#^Contribute equally to this study

**Correspondence**

**Dr. Hui Ding,**

NO.9, Huangpujiang Road,

Huangdao district, Qingdao 266555,

PR China.


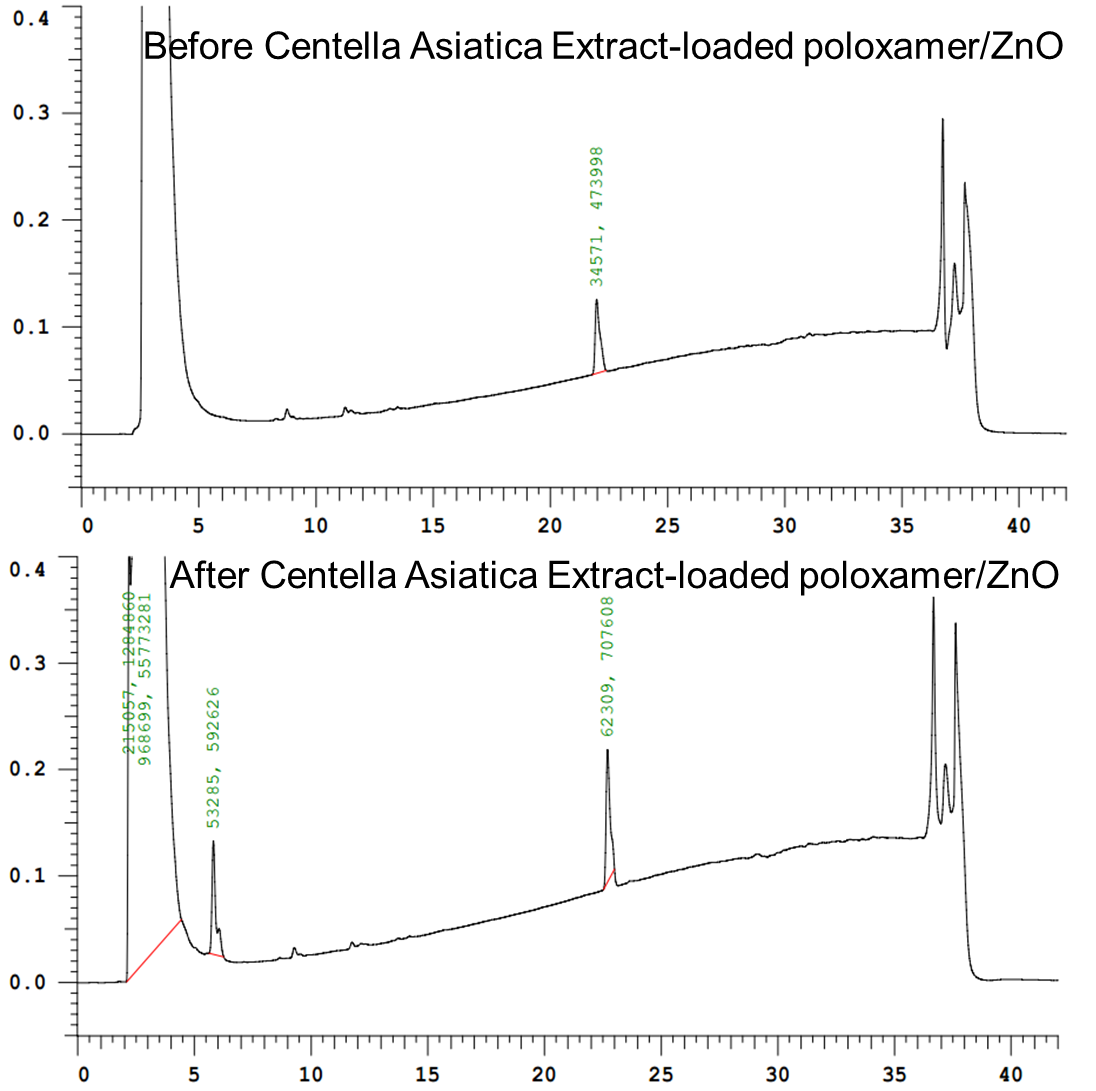


Figure S1. HPLC data of before and after encapsulation of Centella Asiatica Extract-loaded poloxamer/ZnO nanocomposites.
